# *Oligophrenin-1 (OPHN1)*, a Gene Involved in X-Linked Intellectual Disability, Undergoes RNA Editing and Alternative Splicing during Human Brain Development

**DOI:** 10.1371/journal.pone.0091351

**Published:** 2014-03-17

**Authors:** Sabina Barresi, Sara Tomaselli, Alekos Athanasiadis, Federica Galeano, Franco Locatelli, Enrico Bertini, Ginevra Zanni, Angela Gallo

**Affiliations:** 1 Molecular Medicine Laboratory, Neurosciences Department, Bambino Gesù Children's Hospital IRCCS, Rome, Italy; 2 RNA Editing Laboratory, Oncohaematology Department, Bambino Gesù Children's Hospital IRCCS, Rome, Italy; 3 Instituto Gulbenkian de Ciência, Oeiras, Portugal; 4 Università di Pavia, Pavia, Italy; CNRS UMR7275, France

## Abstract

*Oligophrenin-1* (*OPHN1*) encodes for a Rho-GTPase-activating protein, important for dendritic morphogenesis and synaptic function. Mutations in this gene have been identified in patients with X-linked intellectual disability associated with cerebellar hypoplasia. ADAR enzymes are responsible for A-to-I RNA editing, an essential post-transcriptional RNA modification contributing to transcriptome and proteome diversification. Specifically, ADAR2 activity is essential for brain development and function. Herein, we show that the *OPHN1* transcript undergoes post-transcriptional modifications such as A-to-I RNA editing and alternative splicing in human brain and other tissues. We found that *OPHN1* editing is detectable already at the 18^th^ week of gestation in human brain with a boost of editing at weeks 20 to 33, concomitantly with *OPHN1* expression increase and the appearance of a novel *OPHN1* splicing isoform. Our results demonstrate that multiple post-transcriptional events occur on *OPHN1*, a gene playing an important role in brain function and development.

## Introduction

The human *Oligophrenin-1 (OPHN1)* gene is located on chromosome Xq12, encompasses 25 exons and is translated into a protein of 802 amino acids (corresponding to exons 2–24). *OPHN1* encodes for a Rho-GTPase-activating protein that promotes GTP hydrolysis and regulates the activity of Rho proteins [1]. Rho subfamily members are key mediators of cytoskeletal remodelling, which affects several cellular functions including neuronal cell migration and synaptic morphogenesis [2].

*OPHN1* was first identified in a female patient showing mild intellectual disability and carrying a (X;12)(q11;q15) translocation [1]. Since then, a number of mutations of this gene have been reported in X-linked intellectual disability (XLID) associated with cerebellar hypoplasia [3], [4]. The importance of OPHN1 for brain development/function has also been demonstrated in mice, where *ophn1*-defective neurons show dendritic spine immaturity and alterations in synaptic function [5]. Indeed, oligophrenin-1 downregulates the RhoA/Rho-kinase signalling pathway, repressing its inhibitory activity on synaptic vesicle recycling and AMPAR internalization [6]. Moreover, Ophn1 interacts with Rev-erbα, an orphan nuclear receptor involved in the murine hippocampus circadian clock regulation, inducing its localization in dendrites and spines [7].

In mammals, genes are highly processed after transcription by different post-transcriptional mechanisms, such as alternative splicing and RNA editing. However, while splicing implies a cut-and-paste mechanism of nucleotide portions encoded by DNA, RNA editing alters the RNA sequences generating molecules different from those coded by DNA [8]–[10]. The most frequent type of RNA editing in mammals is the deamination of adenosines (A) into inosines (I) within double-stranded RNAs (dsRNAs), through the action of ADAR (adenosine deaminase acting on RNA) enzymes [8]–[10]. ADARs recognize dsRNA structures through their RNA binding domains (RBDs) at the amino terminus and convert adenosine into inosine by their highly conserved deaminase domain (DM) at the carboxy terminus [8]–[10]. In mammals, there are three ADAR proteins: ADAR1-3. ADAR1 and ADAR2 are active enzymes expressed in many different tissues, while ADAR3 seems to be inactive and expressed exclusively in the brain [8]–[10]. Usually, editing at a specific site is not 100% efficient; therefore, both the edited and the unedited RNA variants coexist within a cell. Since inosine is recognised as guanosine by both splicing and translation machineries, RNA editing has the potential to alter splicing sites and amino acid codons, increasing the number of RNA and protein isoforms [9].

Bioinformatics studies and next generation sequencing have revealed that in humans the majority of A-to-I RNA editing events (corresponding to A-to-G changes in the cDNA) lay within non-coding portions of pre-mRNAs, such as introns and untranslated regions (UTRs) [11]–[14]. Specifically, it has been shown that RNA editing events are frequent in inverted Alu repeats, usually folded in dsRNA structures, located in introns and UTRs [9], [15].

A-to-I RNA editing plays an essential role in brain development in both *Drosophila* and mammals [16]–[18]. In *Drosophila*, several genes involved in synaptic vesicle release machinery are targets of the dADAR enzyme (*e.g. endophilin A*) [19]. In mammals, ADAR2-mediated editing is crucial for the activity of many proteins expressed in the Central Nervous System (CNS) and important for normal brain function, such as FLNA (Filamin A), CYFIP2 (cytoplasmic FMR1 interacting protein 2), GluR-B (α-amino-3-hydroxy-5-methylisoazol-4-propionate (AMPA)-receptor subunit) and 5HT2C (serotonin receptor) [20]–[22]. Furthermore, *Adar2* ^−/−^ knockout mice become prone to seizures and die at a post-natal stage due to the editing loss at the Q/R site within the *GluR-B* transcript [17]. Notably, it has been shown that alterations of ADAR2 editing activity are involved in several human diseases affecting the CNS [10], [23].

In the present study, we demonstrate that *OPHN1*, a Rho-GTPase-activating protein essential for neuronal development and synaptic function, undergoes post-transcriptional modification events such as A-to-I RNA editing and alternative splicing during human brain development.

## Materials and Methods

### Cell lines

Human astrocytoma cell lines U118 MG (HTB-15TM) and U87 MG (HTB-14TM) were obtained from American Type Culture Collection (ATCC) and kindly supplied by Dr. S. Galardi (University of Tor Vergata, Rome, Italy). U118 and U87 cell lines stably overexpressing the active or the inactive ADAR2 enzyme were generated as previously reported [24]. U118 and U87 cells stably silenced for ADAR1 enzyme were generated using the BLOCK-iT Inducible Pol II miR RNAi Expression Vector Kit with EmGFP (K4939-00 - Invitrogen, Carlsbad, CA, USA), according to the manufacturer's instructions. All cell lines were grown in Dulbecco's modified Eagle's medium supplemented with 10% fetal calf serum (10270 - Gibco-Life Technologies, Glasgow, UK) plus antibiotics, at 37°C in 5% CO2.

### Tissues

Human normal spinal cord (NICHD, Brain and tissue bank, USA), human normal brain (obtained from a pediatric patient undergoing focal brain resection for head injury sequelae) and human normal skin (obtained from a biopsy) tissues were used to compare the cDNA sequence to its corresponding gDNA. Total RNA from pools of different subjects was also used for RNA editing analysis. Specifically, we used total RNA from human adult brain (a pool of 3 individuals, AM6000 - Ambion-Life Technologies), human fetal brain (a pool of 2 individuals, 18^th^ gestation week, 540157 - Stratagene-Agilent, La Jolla, CA, USA), human fetal brain (a pool of 59 individuals, 20^th^–33^rd^ gestation week, 636526 - Clontech, Palo Alto, CA, USA), human cerebellum (a pool of 10 individuals, 636535 - Clontech), human kidney (a pool of 3 individuals, AM6000 - Ambion-Life Technologies), and human thyroid (a pool of 3 individuals, AM6000 - Ambion-Life Technologies).

### RNA isolation, reverse transcription (RT-PCR), sequencing and RNA editing analysis

Total RNA and genomic DNA were isolated using TRIzol reagent (Invitrogen) according to the manufacturer's instructions. Each RNA sample was DNase treated (Recombinant DNase I (RNase free), AM2235 - Ambion) and quantified by NanoDrop 2000 (Thermo Scientific, Philadelphia, PA, USA). cDNAs were generated by ImProm-II Reverse Transcription System (A3800 - Promega, Madison, WI, USA) or Superscript II reverse transcriptase (18064 - Invitrogen) using random hexamers or transcript-specific oligonucleotides. Three independent RT-PCRs (reverse transcriptase-polymerase chain reactions) were performed for each sample. The cDNAs were amplified by PCR reactions using Expand high fidelity Plus PCR System (03300226001 - Roche, Sydney, Australia). Direct sequencing (ABI 3500 Genetic Analyzer, Applied Biosystems-Life Technologies) was performed on cDNA pools and editing levels were calculated as previously described [25], [26]. Briefly, editing was quantified dividing the height of the G peak by the sum of the A and G peaks of the analyzed site. All primer sequences used for these studies are shown in Table S1 see File S1.

### Analysis of mRNA expression levels

Gene-specific exon-exon boundary PCR products (TaqMan gene expression assays, Applied Biosystems) were measured by means of a PE Applied Biosystems PRISM 7700 sequence detection system during 40 cycles. *β-actin* mRNA was used for normalization and relative quantification of gene expression was performed according to the 2-ΔΔCt method. Expression levels were represented in arbitrary units calculated as a relative-fold increase compared to the control sample arbitrarily set to 1. Quantitative RT-PCRs were repeated in triplicates from at least two independent experiments.

The primers were supplied by Applied Biosystems: *OPHN1*, ID Hs00609994_m1; *ADAR1*, ID Hs00241666_m1; *ADAR2*, ID Hs00953730_m1; *β-actin*, ID Hs99999903_m1. All the qRT-PCR data was also confirmed using the SYBR green method (Invitrogen) (data not shown).

### Statistical analysis

A non-paired Student's T-test was used for statistical evaluation. A two-sided *p* value lower than 0.05 was accepted to indicate statistical significance.

### Ethics Statement

The study was revised and approved by the local Institutional Review Board (IRB) of Bambino Gesù Children's Hospital of Rome, regulating the use of human samples for experimental studies. Informed written consent to use the biological samples for research purposes was obtained from all the patients' parents.

## Results

### New A-to-I editing events in *OPHN1* pre-mRNA

In order to identify possible A-to-I RNA editing events within the *OPHN1* transcript, we interrogated the available editing database (http://darned.ucc.ie) [27]. No editing site has been detected within the human *OPHN1* coding region, as also confirmed by direct sequencing of the *OPHN1* cDNA (exon 2–24) in human brain and spinal cord tissues (data not shown). We only detected a single G/A change in the exon 2 of the *OPHN1* genomic sequence and in the corresponding cDNA of our samples, already reported as a single nucleotide polymorphism (SNP) (rs41303733, nucleotide position ChrX:67652748 in GRCh37.p10, corresponding to the V39I amino acid change) in different databases (www.ensembl.org; https://www.ncbi.nlm.nih.gov/SNP/).

**Figure 1:**
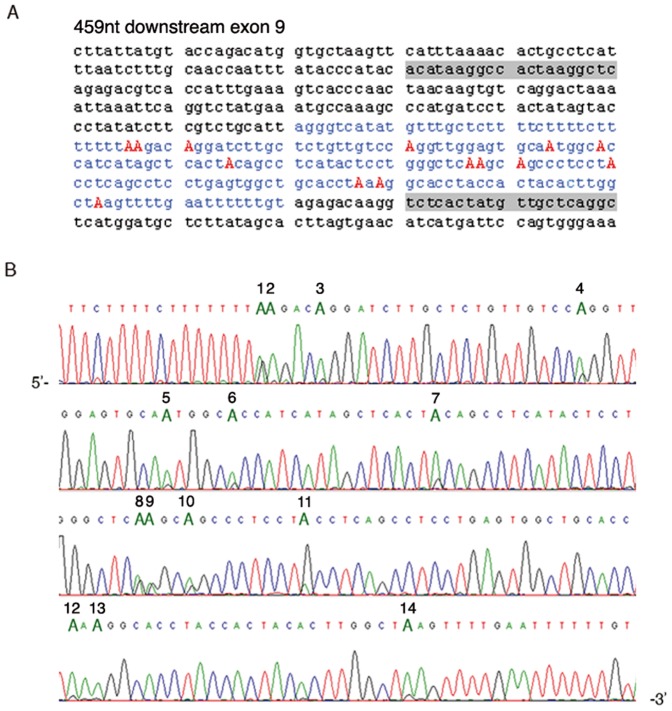
Editing events in the human *OPHN1* transcript. (**A**) Sequence of an intronic portion of *OPHN1* pre-mRNA (intron 9–10), with AluJo region in blue and adenosines undergoing editing in red capital letters. In grey, primers used for PCR amplifications (AluJo Fw and AluJo Rev, Table S1, see File S1). (**B**) Chromatogram of the AluJo region isolated from human brain cDNA, showing the newly identified editing sites, named as 1 to 14 and represented as a double peak of adenosine (green) and guanosine (black). The same positions (1–14) in the corresponding human brain gDNA sequence are only adenosine (Figure S1 in File S1).

**Figure 2:**
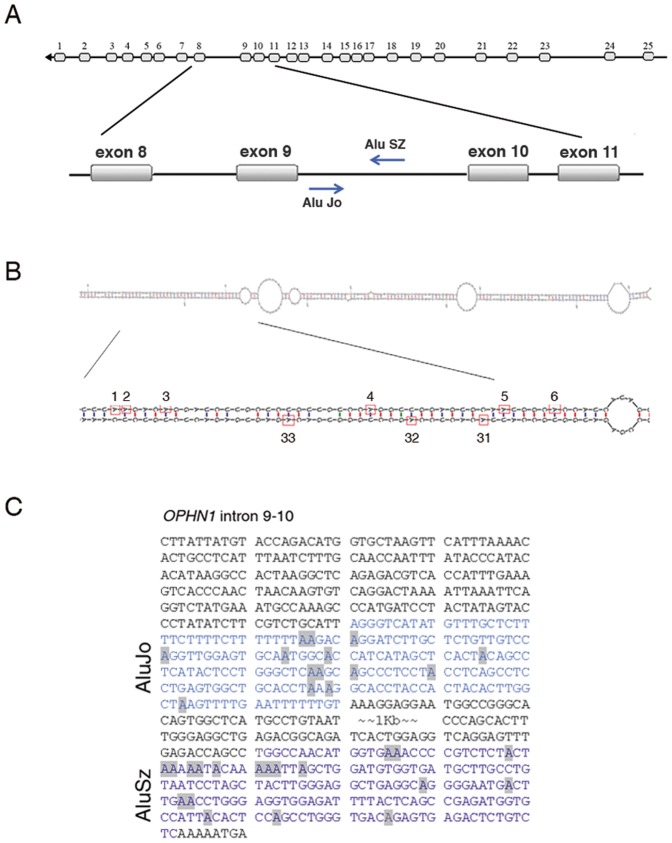
Human *OPHN1* gene and transcript organization. (**A**) Schematic representation of *OPHN1* gene with exons represented as light grey rectangles and introns as black lines. Blue arrows indicate AluJo and AluSz within intron 9–10. (**B**) The predicted dsRNA secondary structure (by Zuker algorithm) formed by the AluJo and AluSz sequences. In detail a portion of the dsRNA structure with red rectangles indicating the edited adenosines. (**C**) *OPHN1* pre-mRNA sequence of the intron 9–10, showing the AluJo and AluSz regions and the 33 adenosines that undergo editing in grey boxes.

Then we looked for inverted Alu repeats with low degree of divergence as previously described [11]. Potential editing sites were predicted *in silico* in the *OPHN1* pre-mRNA within an Alu element (AluJo) (intron 9–10), 459 nt downstream of exon 9 (Figure 1A). The AluJo cDNA sequence generated from human brain tissue, using *OPHN1* intronic specific primers (Table S1 in File S1), showed 14 A-to-G changes representing new potential editing sites (Figure 1B). Indeed, no A-to-G changes were found at the same positions in the corresponding AluJo genomic sequence (Figure S1 in File S1). It is well-established that ADARs need to bind a dsRNA structure to edit. Indeed, we identified a complementary inverted Alu repeat (AluSz) within the same intron 9–10, ∼1 Kb downstream AluJo (Figure 2A). AluSz can base-pair with the AluJo region creating a long dsRNA structure (Figure 2B) with a ΔG = −260 free energy, as predicted by the Zuker algorithm [28]. Actually we found that also AluSz undergoes editing, with a total of 33 new editing events within both AluJo and AluSz sequences, as analysed by PCR sequencing reactions of human brain cDNA and gDNA (Figure 2C and data not shown).

**Figure 3:**
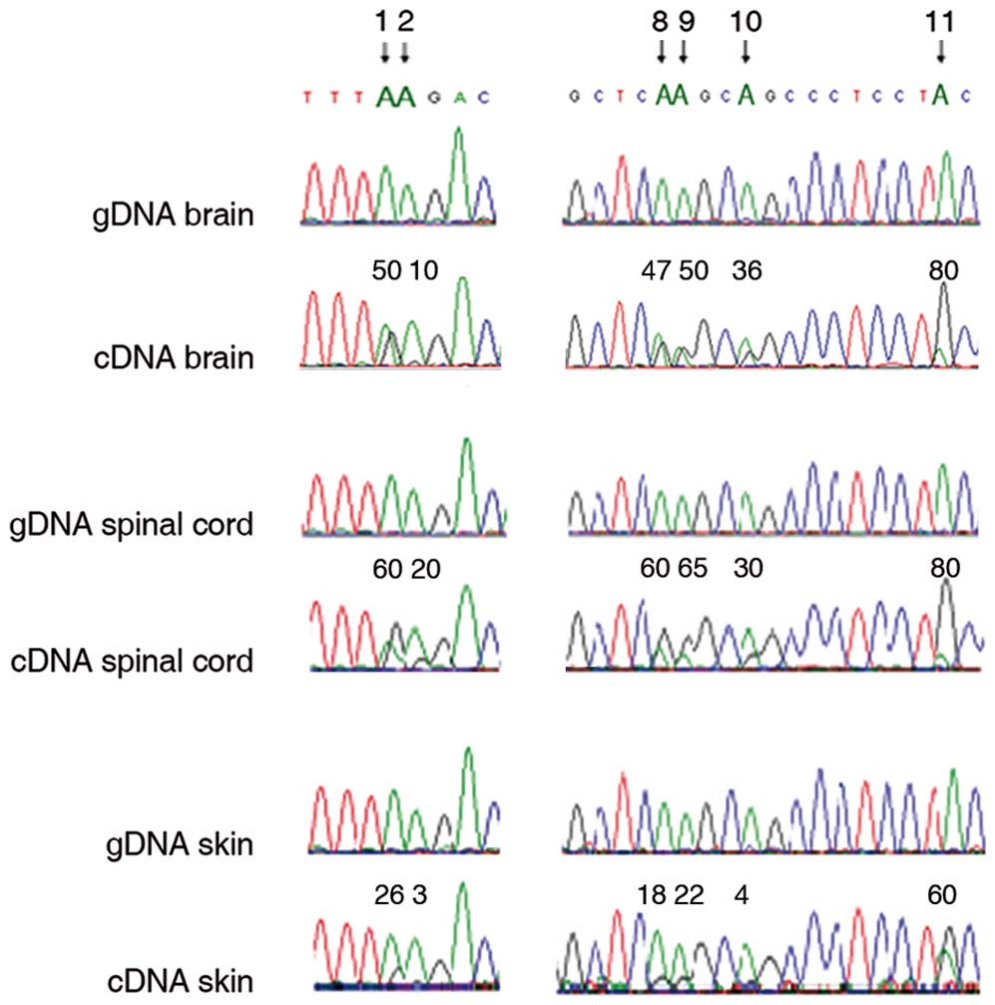
Partial sequence chromatograms of the *OPHN1* intron (AluJo) isolated from different human tissues (brain, spinal cord, skin). For each tissue both the gDNA and the corresponding cDNA are shown. Arrows indicate selected edited positions (site 1, 2, 8, 9, 10 and 11) and the corresponding editing levels of the sequence chromatograms are reported above each site as percentages (%). Editing appears as a double peak of adenosine (green) and guanosine (black) in cDNA sequences, whereas only adenosines are present in the gDNA. A representative experiment out of three is shown. Editing levels at all the AluJo editing sites as found in human brain, spinal cord, skin as well as kidney and thyroid are reported in Table 1.

Although OPHN1 plays an essential role in neuronal plasticity, it is expressed in several tissues [1]. Therefore, we searched for *OPHN1* editing events in randomly selected human tissues: spinal cord, skin, kidney and thyroid. All the tissues analysed showed the presence of editing events in the AluJo, even if the percentages of RNA editing levels vary between the different tissues, with the highest values observed in human brain and spinal cord and the lowest observed in skin and thyroid (Figure 3 and Table 1). However, despite differences in editing levels among these tissues (Table 1), the general editing pattern was preserved, with some hot-spot sites (site 1-8-9-11) always showing high editing values in all the tissues analysed (Table 1).

**Table 1:** *OPHN1* RNA editing levels in human tissues.

*OPHN1*	Brain	Spinal Cord	Skin	Kidney	Thyroid
site 1	46.76 (±0.83)	52.95 (±2.96)	20.32 (±4.01)	33.03 (±0.9)	24.25 (±0.85)
site 2	17.14 (±2.27)	20.39 (±2.02)	6.74 (±3.39)	16.16 (±1.13)	8.48 (±1.24)
site 3	15.91 (±1.53)	17.92 (±2.1)	3.30 (±3.3)	6.93 (±0.12)	9.17 (±0.47)
site 4	22.85 (±4.02)	21.02 (±4.03)	5.46 (±3.67)	8.22 (±1.18)	0
site 5	17.79 (±1.49)	19.64 (±2.02)	1.69 (±1.69)	8.07 (±0.98)	3.26 (±3.26)
site 6	26.56 (±1.82)	31.52 (±2.27)	1.91 (±1.9)	8.16 (±0.7)	13.58 (±1.73)
site 7	26.91 (±1.21)	29.48 (±1.58)	7.54 (±3.78)	8.16 (±0.7)	13.58 (±1.73)
site 8	51.45 (±1.69)	54.19 (±4.37)	23.61 (±3.23)	41.62 (±1.21)	38.03 (±1.4)
site 9	54.35 (±3.09)	68.55 (±4.51)	27.17 (±2.83)	40.26 (±0.29)	37.42 (±0.05)
site 10	35.31 (±1.46)	32.78 (±4.65)	7.89 (±4.34)	10.12 (±0.16)	15.74 (±1.78)
site 11	79.80 (±1.17)	80.14 (±2.61)	54.92 (±6.19)	56.21 (±0.32)	47.76 (±0.91)
site 12	7.29 (±2.23)	2.06 (±2.75)	2.67 (±2.67)	0	1.94 (±1.94)
site 13	4.08 (±2.64)	10.18 (±3.80)	0	0	0
site 14	1.69 (±1.69)	0	0	0	0

RNA editing levels of the AluJo sequence in *OPHN1* pre-mRNA (sites 1–14) in human adult brain, spinal cord, skin, kidney and thyroid tissues. All the editing percentages are expressed as mean ± s.e.m. (n = 3).

### Both ADAR1 and ADAR2 enzymes edit the *OPHN1* transcript

In order to identify which ADAR (ADAR1 and/or ADAR2) was responsible for the editing events found in the *OPHN1* transcript, we took advantage of the astrocytoma cell lines (U118 and U87) available in our laboratory, in which we stably modulate the expression of the ADAR enzymes. Of note, previous studies have already reported that *OPHN1* is expressed in astrocytoma/GBM tumors [29].

Astrocytoma cell lines show RNA editing activity mediated by ADAR1 (as observed at the hotspot ADAR1-specific editing site within the miniB13 transgene [17]) and a low/null ADAR2 editing activity (as observed at the GluR-B Q/R ADAR2-specific editing site within the miniB13 transgene [17]) (Figure S2 in File S1), as previously reported [24], [25], [30]. In view of the above data, we investigated the editing profile of *OPHN1* in these cell lines either stably silenced for ADAR1 (70–80% at protein level, Figure S3 in File S1) or stably overexpressing ADAR2 or its inactive version (ADAR2 E/A) [24], [25]. In our cell lines, we found that both ADAR enzymes are able to edit *OPHN1*. Specifically, we found that the sites 3, 4 and 10 are preferentially edited by ADAR2 (Table 2 upper panel, sites in bold) and the sites 7, 8 and 9 are preferentially edited by ADAR1 (Table 2 lower panel, sites in bold). The sites 1 and 11 (represented in bold and underlined in the Table 2) are significantly modulated by both ADAR enzymes in U118 and U87 cell lines (Table 2).

**Table 2:** Editing profile of *OPHN1* in astrocytoma cell lines overexpressing ADAR2 or silenced for ADAR1.

Astrocytoma cell lines overexpressing ADAR2						
*OPHN1*	U118 E/A	U118 Ad2	*p* values	U87 E/A	U87 Ad2	*p* values
**site 1**	19.96 (±3.64)	35.60 (±2.44)	**0.0287**	14.63 (±3.97)	30.61 (±1.41)	**0.0053**
site 2	2.10 (±2.10)	0	0.2856	2.71 (±1.66)	3.88 (±2.57)	0.7120
**site 3**	2.87 (±2.87)	21.41 (±3.72)	**0.0141**	1.54 (±1.54)	13.41 (±2.03)	**0.0016**
**site 4**	3.86 (±3.86)	46.69 (±5.76)	**0.0024**	5.39 (±2.28)	29.73 (±4.82)	**0.0019**
site 5	6.30 (±3.20)	18.26 (±3.44)	0.0576	3.01 (±1.94)	6.69 (±3.07)	0.3391
site 6	7.02 (±3.66)	14.75 (±1.91)	0.1445	2.33 (±2.33)	8.82 (±2.42)	0.1006
site 7	8.50 (±4.25)	17.92 (±1.56)	0.0657	12.28 (±1.49)	16.40 (±1.22)	0.0645
site 8	21.04 (±4.26)	16.13 (±2.46)	0.3347	17.29 (±4.34)	18.22 (±3.08)	0.8653
site 9	23.96 (±5.21)	17.18 (±1.08)	0.1955	20.64 (±4.49)	21.57 (±3.06)	0.8691
**site 10**	8.38 (±4.68)	50.75 (±8.40)	**0.0107**	6.56 (±2.15)	27.31 (±6.53)	**0.0166**
**site 11**	39.38 (±8.11)	69.41 (±5.91)	**0.0274**	35.75 (±6.64)	59.44 (±2.92)	**0.0143**
site 12	0	9.42 (±3.77)	0.1163	0	7.96 (±3.40)	0.0776
site 13	0	0	nd	0	0	nd
site 14	0	0	nd	0	0	nd

Astrocytoma cell lines silenced for ADAR1						
*OPHN1*	U118 scr	U118 siAd1	*p* values	U87 scr	U87 siAd1	*p* values
**site 1**	24.27 (±0.95)	10.18 (±3.53)	**0.021**	23.70 (±3.06)	7.72 (±2.06)	**0.0105**
site 2	5.23 (±1.96)	1.91 (±1.91)	0.2705	3.69 (±2.14)	0	0.2052
site 3	0	0	nd	0	3.08 (±3.08)	0.2856
site 4	0	0	nd	0	1.74 (±1.74)	0.2856
site 5	0	0	nd	2.61 (±2.61)	0	0.4366
site 6	3.41 (±3.41)	0	0.3559	5.0 (±3.50)	0	0.2815
**site 7**	11.51(±1.26)	0.97 (±0.97)	**0.0006**	13.29 (±0.39)	3.18 (±1.66)	**0.0010**
**site 8**	19.87 (±2.87)	9.06 (±3.13)	**0.0437**	25.28 (±0.70)	3.57 (±3.57)	**0.0009**
**site 9**	23.46 (±2.58)	11.37 (±3.95)	**0.0429**	27.59 (±2.41)	4.40 (±4.40)	**0.0042**
site 10	3.68 (±2.25)	0	0.15	5.0 (±3.11)	6.33 (±3.18)	0.7821
**site 11**	48.61 (±3.55)	19.93 (±1.42)	**0.004**	50.6 (±1.62)	25.95 (±3.34)	**0.0008**
site 12	0	0	nd	0	0	nd
site 13	0	0	nd	0	0	nd
site 14	0	0	nd	0	0	nd

Editing analysis of OPHN1 pre-mRNA (AluJo, sites 1–14) in U118 and U87 astrocytoma cell lines overexpressing ADAR2 or silenced for ADAR1. The sites preferentially edited by a specific enzyme are indicated in bold and the sites edited by both ADARs are in bold and underlined. The sites 7-8-9 are mainly edited by ADAR1 (as a strong decrease of editing values at these sites is visible after ADAR1 silencing, whilst ADAR2 overexpression does not cause modifications). The sites 3-4-10 are specifically edited by ADAR2 (as a significant increase of editing percentages at these sites is visible in ADAR2 overexpressing cells, whilst no change appears after ADAR1 silencing). Sites 1 and 11 are significantly modulated by both the ADAR enzymes. The RNA editing levels are expressed as percentages (mean ± s.e.m, n = 3). nd = not determined.

### ADAR2-mediated editing and expression of *OPHN1* are correlated *in vitro*

**Figure 4:**
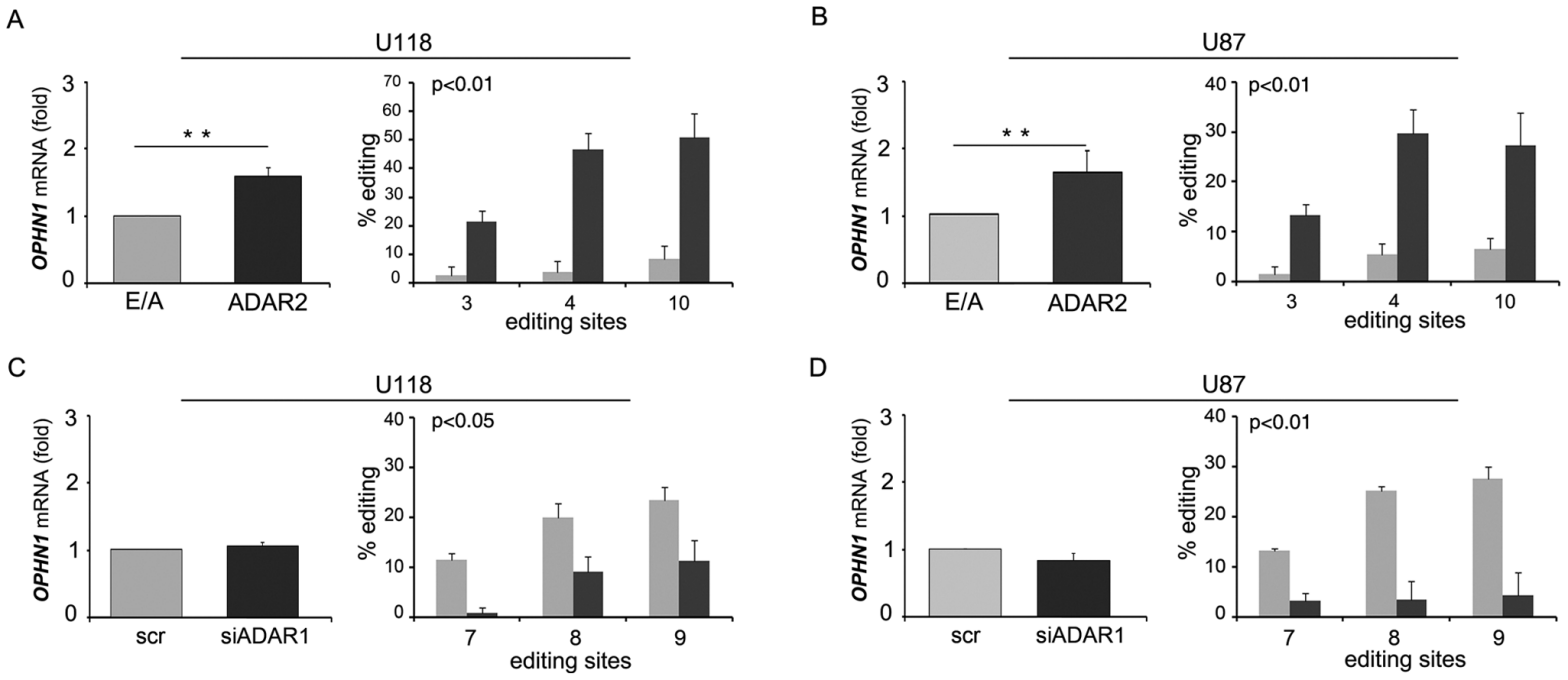
*OPHN1* RNA editing and expression in astrocytoma cell lines. (**A**) *OPHN1* mRNA expression (left panel) and editing levels at ADAR2-specific sites (right panel) in U118 ADAR2 E/A (light grey) and U118 ADAR2 (dark grey) cells. The expression levels were calculated as a relative-fold increase compared to inactive ADAR2 (E/A) arbitrarily set to 1. Each sample was normalized to *β-actin* mRNA levels. Mean ± s.d. (n = 3), ** *p*<0.01. (**B**) The same experiments showed in (A) were performed in the U87 cell line. (**C**) *OPHN1* mRNA expression (left panel) and editing levels at the ADAR1-specific sites (right panel) in U118 scramble (light grey) and U118 siADAR1 (dark grey) cells. The expression levels of the samples were calculated as a relative-fold increase compared to the scramble arbitrarily set to 1. Each sample was normalized to *β-actin* mRNA levels. Mean ± s.d. (n = 3). (**D**) The same experiments showed in (C) were performed in the U87 cell line.

We monitored *OPHN1* expression and editing in both U118 and U87 cells upon ADARs modulation. Comparing astrocytoma cell lines overexpressing active (ADAR2) and inactive ADAR2 (ADAR2 E/A), we observed a significant OPHN1 increase (at both mRNA and protein levels) only when the active ADAR2 was present (Figure 4A–B, Figure S4 in File S1 and data not shown), along with a significant increase of editing values at the ADAR2-specific editing sites (sites 3-4-10) (Figure 4A–B and Table 2).

Notably, no statistical differences were detected in *OPHN1* levels upon ADAR1 silencing (Figure 4C–D), despite a significant fluctuation of editing values at the ADAR1-specific sites (sites 7-8-9) was present (Figure 4C–D and Table 2).

### *OPHN1* editing and expression significantly increase during human brain development

**Figure 5:**
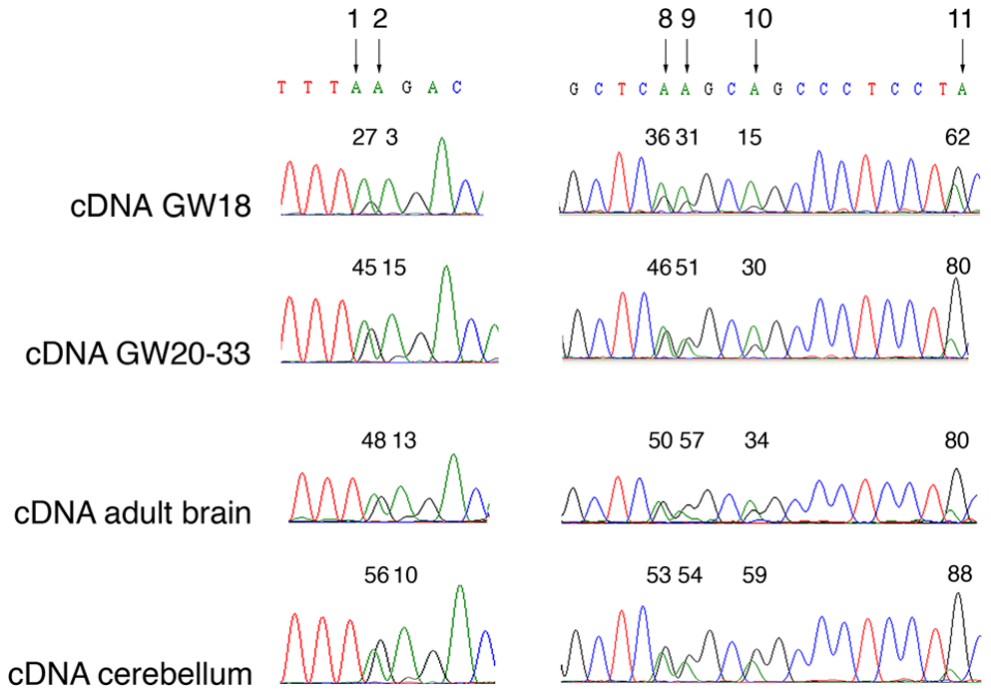
Editing analysis of *OPHN1* (AluJo) during human brain development and in the cerebellum. Partial sequence chromatograms of the AluJo isolated from a pool of fetal brains at the 18^th^ gestation week (GW18), a pool of fetal brains at the 20^th^–33^rd^ gestation weeks (GW20–33), a pool of adult brains and a pool of cerebella are shown. Selected sites and the corresponding editing values (%) of one out of three representative experiments are shown. Editing percentages of sites 1–14 are reported in Table 3.

Due to the importance of *OPHN1* for brain development and cerebellar function, we investigated whether - and at which extent - editing events occur also in human fetal brain and cerebellum. To this aim, we sequenced the AluJo cDNA in fetal brain at an early stage of development (18^th^ gestation week, GW18, pool of 2 subjects), fetal brain at a later stage of development (20^th^–33^rd^ gestation week, GW20–33, pool of 59 subjects), adult brain (pool of 3 subjects) and cerebellum (pool of 10 subjects). Editing analysis showed that *OPHN1* undergoes A-to-I editing events in fetal brain already at GW18 (Figure 5 and Table 3). Interestingly, at GW20–33 the editing activity increases at all sites, with values comparable to those observed in the adult brain (Table 3). In cerebellum we detected editing levels similar or even higher (at sites 3-4-5-10-12) to those found in adult brain, suggesting that the highest ADAR activity over *OPHN1* occurs in this specific brain area (Figure 5 and Table 3).

**Table 3:** OPHN1 RNA editing levels during human brain development and in cerebellum.

*OPHN1*	GW18	GW20–33	Adult brain	Cerebellum
site 1	20.84 (±5.33)	44.45 (±1.06)	46.76 (±0.83)	50.51 (±3.11)
site 2	5.25 (±0.57)	11.36 (±2.63)	17.14 (±2.27)	12.83 (±1.2)
site 3	4.7 (±2.46)	11.6 (±0.53)	15.91 (±1.53)	21.07 (±0.82)
site 4	9.4 (±0.91)	13.73 (±0.26)	22.85 (±4.02)	42.95 (±6.43)
site 5	3.17 (±1.99)	19.19 (±1.32)	17.79 (±1.49)	25.30 (±2.28)
site 6	6.95 (±3.49)	26.49 (±0.63)	26.56 (±1.82)	27.23 (±3.64)
site 7	5.94 (±3.02)	26.23 (±0.43)	26.91 (±1.21)	24.38 (±1.54)
site 8	30.92 (±7.35)	46.32 (±0.55)	51.45 (±1.69)	48.68 (±3.58)
site 9	39.37 (±6.55)	54.86 (±1.78)	54.35 (±3.09)	49.27 (±2.65)
site 10	9.04 (±5.17)	30.59 (±0.29)	35.31 (±1.46)	50.30 (±6.67)
site 11	61.76 (±2.76)	81.43 (±0.55)	79.80 (±1.17)	84.46 (±2.1)
site 12	2.19 (±2.19)	4.05 (±2.03)	7.29 (±2.23)	17.33 (±1.76)
site 13	8.02 (±0.59)	9.15 (±1.63)	4.08 (±2.64)	6.67 (±6.7)
site 14	2.63 (±2.63)	8.29 (±0.69)	1.69 (±1.69)	6.77 (±6.7)

RNA editing levels (%) of *OPHN1* pre-mRNA (AluJo, sites 1–14) in human fetal brain 18^th^ gestation week (GW18), fetal brain 20^th^–33^rd^ gestation weeks (GW20–33), adult brain and cerebellum are expressed as mean ± s.e.m (n = 3).

**Figure 6:**
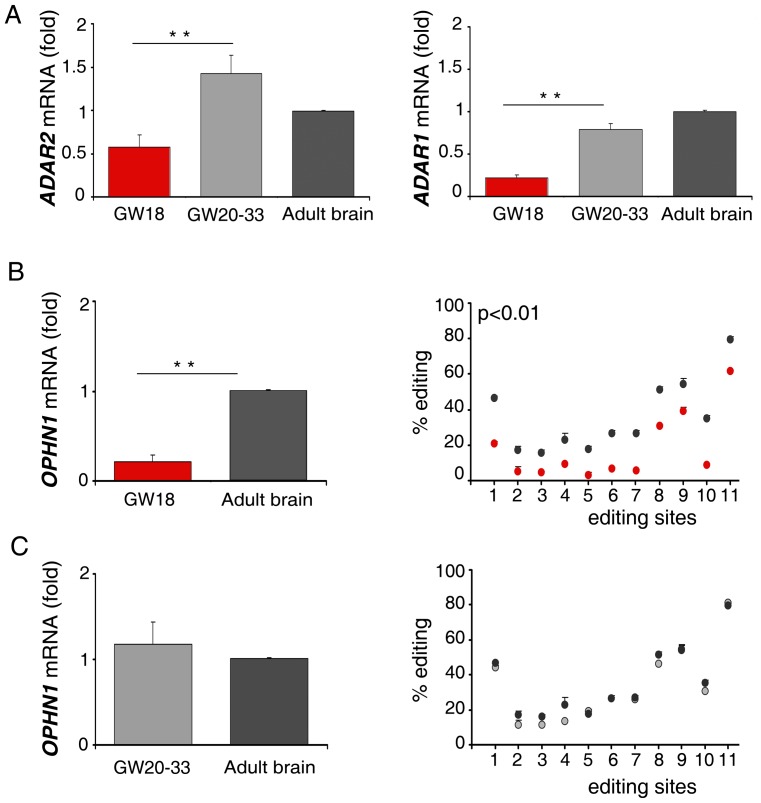
*OPHN1* RNA editing and expression during human brain development. (**A**) *ADAR2* (left panel) and *ADAR1* (right panel) expression analysis in the early (GW18, in red) and late (GW20–33, in light grey) stages of brain development and in adult brain (in dark grey). (**B**) *OPHN1* expression (left panel) and editing profile (right panel) in human GW18 fetal brain (in red) compared to adult brain (in dark grey). Editing percentages are expressed as mean ± s.e.m. (n = 3), *p*<0.01 at all the editing sites, with the exception of the site 4 in which *p*<0.05 (n = 3). (**C**) *OPHN1* expression (left panel) and editing profile (right panel) in human GW20–33 fetal brain (in light grey) compared to adult brain (in dark grey). Mean ± s.e.m. (n = 3). The mRNA levels of the samples in A, B, C were calculated as a relative-fold increase compared to the adult brain arbitrarily set to 1. Each sample was normalized to *β-actin* mRNA levels. Mean ± s.d. (n = 3), ** *p*<0.01. Red dots represent editing percentages of the GW18, light grey dots represent editing percentages of the GW20–33 and dark grey dots represent editing percentages of adult brain.

Considering the importance of ADARs and OPHN1 during brain development [3], [5], [17], [31] and the significant increase of *OPHN1* editing from the fetal to the adult brain (Table 3), we extended our analysis of *OPHN1* editing/expression performed *in vitro* (Figure 4) to different stages of human brain development (GW18 and GW20–33) compared to the adult brain. We considered all the *OPHN1* editing sites, excluding the ones with editing levels lower than 10% (sites 12–14, Table 1), as it would be unlikely to detect fluctuations at these sites. Both *ADAR1* and *ADAR2* mRNA expression increased significantly from the early to the late stage of brain development (Figure 6A). Moreover, the brain at an early stage (GW18) showed low levels of *OPHN1* expression and editing, when compared to the adult brain (Figure 6B). Interestingly, in the late stage of brain development (GW20–33), we found a boost of *OPHN1* expression and editing, reaching values similar to those found in adult brain (Figure 6C). Altogether, our data indicated that ADARs activity and *OPHN1* editing and expression are correlated during brain development.

We also observed that there is a correlation between *ADAR2* and *OPHN1* editing/expression in cerebellum compared to adult brain. Specifically, *OPHN1* and *ADAR2* expression are higher in the cerebellum when compared to the adult brain (Figure S5 in File S1), with a significant increase of *OPHN1* editing at the ADAR2-specific sites (sites 3-4-10) (Figure S5 in File S1). Differently, no significant differences in *ADAR1* expression or editing at the ADAR1-specific sites (sites 7-8-9) of *OPHN1* were observed (Figure S5 in File S1). These findings indicated that in cerebellum ADAR2 may play a major role over *OPHN1* editing/expression, similarly to what was observed *in vitro* (Figure 4).

### Novel *OPHN1* splicing isoforms in brain

**Figure 7:**
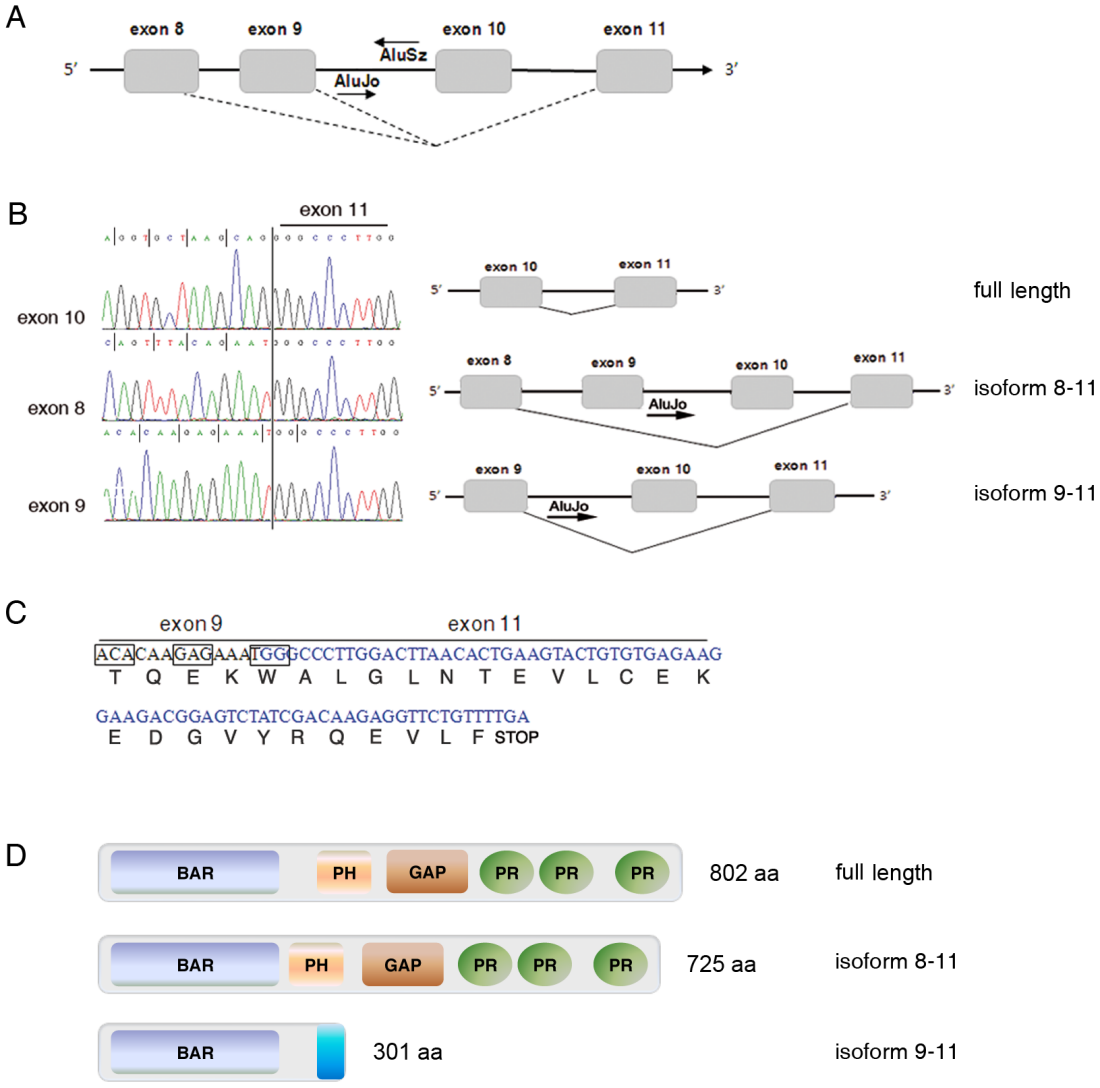
Novel *OPHN1* splicing isoforms. (**A**) Schematic representation of the *OPHN1* pre-mRNA region (exons 8–11) undergoing alternative splicing events. (**B**) Partial sequence chromatograms of cDNAs (left side) with the corresponding schematic representations (right side) of the full length *OPHN1* (upper sequence) and of the two novel *OPHN1* alternative isoforms (middle and bottom sequences). (**C**) The putative protein sequence, derived from the skipping of exon 10 (isoform 9–11) carrying a novel COOH portion, is shown. (**D**) Protein domains of OPHN1 isoforms. The isoform 8–11 carries the BAR and the PH domains at a closer proximity compared to the full length, with possible consequences on protein conformation/activity. The isoform 9–11 carries only the BAR domain. As both the Rho-GTPase activity domain and the PR domains are lost in this isoform, the downstream signalling and the interaction with the actin cytoskeleton could be affected. This isoform displays a new COOH terminal portion of 33 amino acid (shown in blue in the cartoon), with still unknown functions and displaying no homology with known proteins (data not shown). BAR = Bin/Amphiphysin/Rvs, PH = Pleckstrin Homology, GAP = Rho-GTPase Activating Protein, PR = Proline Rich.

We investigated whether *OPHN1* transcript undergoes alternative splicing events in proximity of exons 8–11 in which we identified multiple editing sites (AluJo and AluSz). We found that, in cancer cells (astrocytomas) and brain tissues, *OPHN1* is alternatively spliced in this region (Figure 7A). The first splicing event skips exons 9 and 10, leading to an in-frame mRNA, 231 nucleotides shorter than the full length transcript (called isoform 8–11) (Figure 7B). This alternative transcript might be translated into a shorter protein of 725 amino acids instead of the 802 amino acids full length OPHN1 (Figure 7D). The second splicing event skips exon 10 (101 nt) and leads to an mRNA with an internal frameshift and a downstream stop codon (called isoform 9–11) (Figure 7B). This splicing variant can generate a hypothetical mini-protein of 301 amino acids, carrying the BAR dimerization domain plus 33 amino acids at its COOH terminal that are not present in the full length protein (Figure 7C and D). These two novel variants have not been previously reported or annotated in public databases.

Specific semi-quantitative RT-PCRs (Table S1 in File S1) were performed to detect these two novel *OPHN1* splicing isoforms in human brain tissues at different development stages. We observed that the isoform 8–11 is not expressed at the early stage of brain development (GW18), while it appears at the later stage GW20–33 and further increases in the adult brain (Figure S6 in File S1). The isoform 9–11 was expressed at similar levels at both the early (GW18) and later (GW 20–33) stages of brain development and increased in the adult brain (Figure S6 in File S1).

## Discussion

The *OPHN1* encodes for a Rho-GTPase-activating protein that promotes GTP hydrolysis of Rho subfamily members. OPHN1 carries at the N-terminus a Bin/Amphiphysin/Rvs (BAR) dimerization domain, followed by the Pleckstrin homology (PH) domain that is able to bind membrane lipids and at the C-terminus there are the GAP (Rho-GTPase activating protein) and the Proline Rich (PR) domains. OPHN1 regulates the activity of the Rho proteins, key mediators of several cellular functions including cytoskeleton remodelling, cell migration and synaptic morphogenesis [2], [32]. Specifically, OPHN1 downregulates the RhoA/Rho-kinase signalling pathway, repressing its inhibitory activity on endocytosis and actin-myosin contractility; disruption of Ophn1 in mice results in altered synaptic plasticity and morphology with reduced synaptic vesicle recycling and AMPA receptor internalization [5], [6].

A-to-I RNA editing strongly affects coding and non-coding RNAs by changing adenosine to inosine within RNA, bypassing genomic information [10], [33]. Several studies have connected RNA editing to brain function [17], [34]–[36]; indeed A-to-I RNA editing takes place mostly in the brain compared to other tissues [37], [38], where it modulates the function of several glial/neuronal proteins [34], [39]. Thanks to the recent advances in next generation sequencing methodologies, it has been observed that a huge number of RNAs undergo editing especially in Alu elements often localized within introns and UTRs of transcripts [11], [12], [40].

Herein, we showed that *OPHN1* pre-mRNA, an important transcript for brain function and development, undergoes A-to-I RNA editing within two inverted Alu repeats (AluJo and AluSz), located in intron 9–10. We demonstrated that this transcript undergoes editing in several human tissues, with brain and spinal cord displaying the highest editing percentages (Table 1). Interestingly, cerebellum alone shows editing values resembling the ones observed in the adult brain (Table 3), suggesting that ADAR enzymes are particularly active over this transcript in the cerebellum. Interestingly, *OPHN1* is highly expressed in cerebellum compared to the brain (Figure S5 in File S1) and mutations in this gene are associated with cerebellar hypoplasia [41], [42].

It has been suggested that a possible consequence of multiple RNA editing in long dsRNA structures, as the one found within *OPHN1*, is their destabilization [43]. Indeed, ADAR-mediated editing of an adenosine in an A-U base pair produces a less stable I-U pair, whereas deamination of A:C mismatches leads to more stable I-C pairs. Looking at the best complementary alignment within the *OPHN1* transcript (AluJo-AluSz dsRNA structure) using the Zuker algorithm [28], we found that 70% of the 33 newly detected editing sites were in an A-U pair context (with editing unwinding the dsRNA structure) and that only 15% of the editing events occur in A-C mismatches and 3% in A-G mismatches, with the remaining adenosines located within loops in the dsRNA structure. This indicates that the Adenosines preferentially edited are not randomly distributed along the predicted *OPHN1* dsRNA, but are restricted to positions that alter the dsRNA shape, as previously observed [12]. Changes in dsRNA structures by RNA editing may alter the binding of proteins (ssRNA/dsRNA-binding proteins) involved in the splicing/maturation/localization/amount of transcripts with embedded Alu sequences [44]–[47].

Both ADAR1 and ADAR2 are able to edit OPHN1 transcript (Table 2). Moreover, we reported that editing and expression of *OPHN1* increase when ADAR2 is expressed in astrocytoma cell lines (U118 and U87) (Figure 4). On the contrary, modulation of ADAR1 in the same cells did not affect *OPHN1* mRNA level, despite the significant fluctuation of editing at the ADAR1-specific sites (Figure 4). Additionally, we observed that editing of *OPHN1* progressively increases during brain development concomitantly with its expression (Figure 6).

A link between editing and RNA expression has been suggested by several studies [17], [48]–[50]. However, for only a few of these editing substrates the molecular mechanism involved has been described [51]. Moreover, only recently, it has been shown that the majority of editing events lies within Alu sequences, like the ones reported herein, and it has been suggested that this type of editing is important for gene expression, despite the mechanisms are still not clear [11], [24], [44], [45], [47], [52]. We previously reported that ADAR2-mediated editing increases the CDC14B mRNA/protein levels both *in vitro* and *in vivo* [24]. Herein we show that another transcript (*OPHN1*), known to be involved in human disease, undergoes RNA editing within Alu elements that seems to be linked to its expression in human tissues and cell lines. How ADARs enhance CDC14B and OPHN1 expression is not clear at present and further studies are necessary to address these questions. Of note, A-to-I RNA editing has the potential to alter pre-mRNA specific sequences that are important for RNA splicing and, therefore, for the final amount of the mature RNA [11], [51], [53]. Differences between ADAR1 and ADAR2-mediated effects on *OPHN1* expression could be due to differences among ADAR-specific editing sites that may affect in a different way *OPHN1* maturation.

OPHN1 and ADAR2 are important proteins during brain development [3], [5], [17], [31], [54]. We observed a progressive enrichment of *ADAR2* expression and *OPHN1* expression/editing from the early stage (GW18) to the later stage (GW20–33) of brain development with values similar (*OPHN1* editing/expression, Figure 6C) or even higher (*ADAR2* mRNA expression, Figure 6A) to those found in adult brain. Intriguingly, OPHN1 controls synapse maturation and plasticity by stabilizing AMPA channel receptors [31], whose Ca^2+^ permeability depends on ADAR2 editing activity [17], [55].

In addition to editing, we also identified two novel splicing events that skip either exon 10 (isoform 9–11) or exons 9 and 10 (isoform 8–11). The isoform 8–11 may be translated into a shorter OPHN1 protein isoform with the BAR and the PH domains in a closer proximity compared to the full length protein (Figure 7D). The 9–11 isoform may be translated into a truncated protein carrying only the BAR dimerization domain that could act as a dominant negative protein and compete with the full length OPHN1 [56] (Figure 7D). Indeed, recent studies showed that the N-terminal region of OPHN1 seems to affect its GAP function, suggesting that this part of the protein itself could act as a regulator of GAP activity, either by an autoinhibitory mechanism or by binding of a second inhibitory protein [57]. Notably, we found that these alternative splicing isoforms are differently expressed during brain development. In particular, the 8–11 isoform is absent at the early stage of brain development (GW18), it appears at stage GW20–33 and boosts in adult brain; while the 9–11 isoform expression is already detectable at GW18 and strongly increases in adult brain (Figure S6 in File S1). Of note, a few OPHN1 mutations causing disease map in the BAR domain, which is common to all the splicing variants we identified [58]–[60]. Additional mutations were found also in the PH or GAP domains, which are common to both the full length and the 8–11 OPHN1 variant [41]. Further molecular and biochemical studies will be necessary to disclose how and to which extent RNA editing and the novel alternative splicing isoforms we identified affect OPHN1 protein expression and activity.

In summary, we report that during human brain development *OPHN1* transcript undergoes profound posttranscriptional modifications in brain already at stage GW18, as we observed the presence of *OPHN1* editing (Figure 6B) and the appearance of a new alternative splicing isoform (isoform 9–11). With the progression of brain development (GW20–33), *OPHN1* editing and expression strongly increase reaching values similar to the ones observed in adult brain (Figure 6C). Concomitantly an additional alternative splicing isoform (isoform 8–11) starts to be expressed (Figure S6 in File S1). Notably, synaptogenesis starts at GW20 and neuronal migration is largely completed by GW33 [61], [62].

A recent study in *Drosophila* shows that FMRP (fragile X mental retardation protein 1), responsible for the most common heritable form of intellectual disability, is able to modulate dADAR activity by a direct protein-protein interaction [63], with *Fmr1* mutant flies showing both altered synaptic development and aberrant A-to-I RNA editing [63]. Dysregulation of RNA editing has recently been found in the Prader-Willi syndrome, a neurodevelopmental disorder [35], [64]. Altered RNA editing levels of both the glutamate receptor GRIK2 and the tryptophan hydroxylase TPH2 were also found in the brain of patients with psychiatric disorders [65], [66] and intellectual disability has been reported in patients with ADAR1 mutations [67]. Considering the above, it is intriguing to speculate that altered *OPHN1* editing/splicing could also play an important role in the pathogenesis of intellectual disability.

## Supporting Information

File S1 Includes Figure S1–S6 and Table S1. **Figure S1. Partial sequence chromatogram of the AluJo region (intron 9–10) isolated from the gDNA of a human brain tissue**. The editing sites (1–14) identified in the corresponding cDNA isolated from the same individual (Figure 1) appear as adenosines. **Figure S2. ADAR-mediated RNA editing events within miniB13 transgene**. U118 and U87 astrocytoma cell lines were transiently transfected with miniB13 transgene and editing activity was tested at the GluR-B Q/R site and at the hotspot (+1) site of the miniB13, 48 h post transfection. Percentage (%) of editing is shown. The Q/R site is edited by ADAR2, whilst the hotspot is edited by ADAR1. **Figure S3. ADAR1 expression in U118 and U87 cell lines stably silenced for ADAR1**. (A) *ADAR1* mRNA expression levels of the samples were calculated as a relative-fold increase compared to the untreated cells arbitrarily set to 1. Each sample was normalized to *β-actin*. Mean ± s.d. (n = 3), **p<0.01 (siAd1 versus untreated and scramble). (B) ADAR1 protein levels by immunoblotting of total protein extract from U118/U87 untreated, scramble (scr) and siAdar1 (siAd1) cell lines. No modification of ADAR2 protein level was observed upon ADAR1 silencing in the same cell lines (data not shown)”. **Figure S4. ADAR2 overexpression increases OPHN1 protein levels**. (A) OPHN1 immunoblotting of total protein extract from untreated, ADAR2 and ADAR2 E/A U118 cell lines. A representative experiment out of two is shown. (B) Quantitative densitometric analysis of protein levels. Each sample was normalized to GAPDH and compared to the untreated cells arbitrarily set to 1. Mean ± s.e.m. (n = 2), *p<0.05, ** p<0.01. **Figure S5. RNA editing and expression of** ***OPHN1*** **in cerebellum and adult brain**. (A) *OPHN1* expression in adult brain (dark gray) and cerebellum (black). The mRNA levels of the samples were calculated as a relative-fold increase compared to the adult brain and arbitrarily set to 1. Each sample was normalized to *β-actin* mRNA. Mean ± s.d. (n = 3), **p<0.01. (B) *ADAR2* (left panel) and *ADAR1* (right panel) expression analysis in adult brain (dark gray) and cerebellum (black). The mRNA levels of the samples were calculated as a relative-fold increase compared to the adult brain and arbitrarily set to 1. Each sample was normalized to *β-actin* mRNA. Mean ± s.d. (n = 3), **p<0.01. (C) *OPHN1* ADAR2-editing sites (left panel) and ADAR1-editing sites (right panel) (see Table 3). Mean ± s.e.m. (n = 3), **p<0.01, *p<0.05. **Figure S6. Semi-quantitative RT-PCR analysis of** ***OPHN1*** **alternative isoforms**. (A) PCR reaction of the new alternative splicing isoforms 8–11 and 9–11 during brain development stages (GW18, GW 20–33 and adult), with *GAPDH* used for normalization. (B) Quantitative densitometric analysis of *OPHN1* alternative isoforms 8–11 and 9–11: RNA levels were calculated as a relative-fold increase compared to the adult brain arbitrarily set to 1. **Table S1.** Primers used for sequencing analysis of *OPHN1*.
(PDF)

Methods S1 Includes Supporting Information Methods.
(PDF)
