# Supplementary material for: Oligophrenin-1 (OPHN1), a Gene Involved in X-Linked Intellectual Disability, Undergoes RNA Editing and Alternative Splicing during Human Brain Development
Source: PLoS One. 2014 Mar 17;9(3):e91351. doi: 10.1371/journal.pone.0091351 (PMC3956665; doi:10.1371/journal.pone.0091351)
Supplement: File S1 — Includes Figure S1–S6 and Table S1. Figure S1. Partial sequence chromatogram of the AluJo region (intron 9–10) isolated from the gDNA of a human brain tissue. The editing sites (1–14) identified in the corresponding cDNA isolated from the same individual (Figure 1) appear as adenosines. Figure S2. ADAR-mediated RNA editing events within miniB13 transgene. U118 and U87 astrocytoma cell lines were transiently transfected with miniB13 transgene and editing activity was tested at the GluR-B Q/R site and at the hotspot (+1) site of the miniB13, 48 h post transfection. Percentage (%) of editing is shown. The Q/R site is edited by ADAR2, whilst the hotspot is edited by ADAR1. Figure S3. ADAR1 expression in U118 and U87 cell lines stably silenced for ADAR1. (A) ADAR1 mRNA expression levels of the samples were calculated as a relative-fold increase compared to the untreated cells arbitrarily set to 1. Each sample was normalized to β-actin. Mean ± s.d. (n = 3), **p<0.01 (siAd1 versus untreated and scramble). (B) ADAR1 protein levels by immunoblotting of total protein extract from U118/U87 untreated, scramble (scr) and siAdar1 (siAd1) cell lines. No modification of ADAR2 protein level was observed upon ADAR1 silencing in the same cell lines (data not shown)”. Figure S4. ADAR2 overexpression increases OPHN1 protein levels. (A) OPHN1 immunoblotting of total protein extract from untreated, ADAR2 and ADAR2 E/A U118 cell lines. A representative experiment out of two is shown. (B) Quantitative densitometric analysis of protein levels. Each sample was normalized to GAPDH and compared to the untreated cells arbitrarily set to 1. Mean ± s.e.m. (n = 2), *p<0.05, ** p<0.01. Figure S5. RNA editing and expression of OPHN1 in cerebellum and adult brain. (A) OPHN1 expression in adult brain (dark gray) and cerebellum (black). The mRNA levels of the samples were calculated as a relative-fold increase compared to the adult brain and arbitrarily set to 1. Each sample was normalized to β-actin mR [file pone.0091351.s001.pdf]

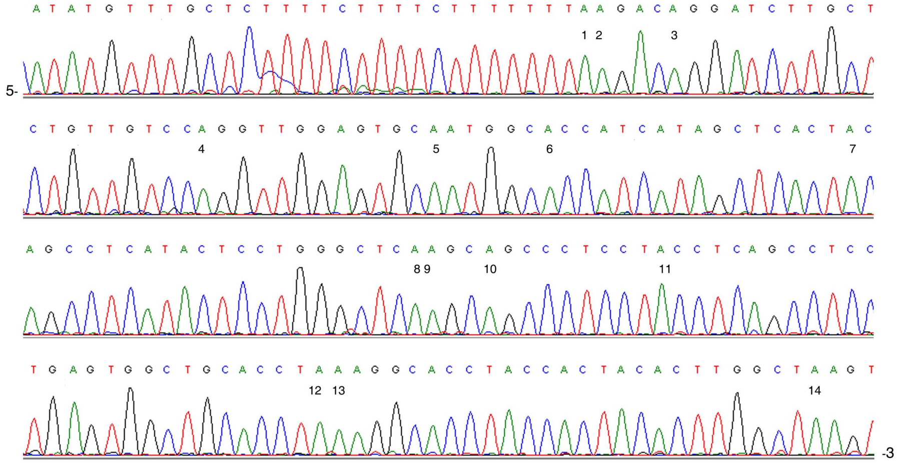
A T A T G T T T G C T C T T T T C T T T T C T T T T T T T A A G A C A G G A T C T T G C T
1 2
3
5-
C T G T T G T C C A G G T T G G A G T G C A A T G G C A C C A T C A T A G C T C A C T A C
4
5
6
7
A G C C T C A T A C T C C T G G G C T C A A G C A G C C C T C C T A C C T C A G C C T C C
8 9
10
11
T G A G T G G C T G C A C C T A A A G G C A C C T A C C A C T A C A C T T G G C T A A G T
12 13
14
-3

Fig. S1

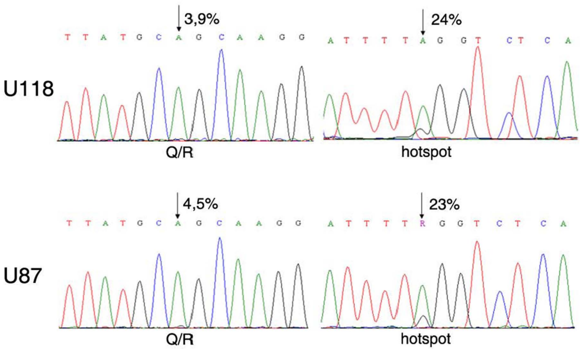

3,9%
T T A T G C A G C A A G G
24%
A T T T T A G G T C T C A
U118
Q/R
hotspot
4,5%
T T A T G C A G C A A G G
23%
A T T T T R G G T C T C A
U87
Q/R
hotspot

Fig. S2

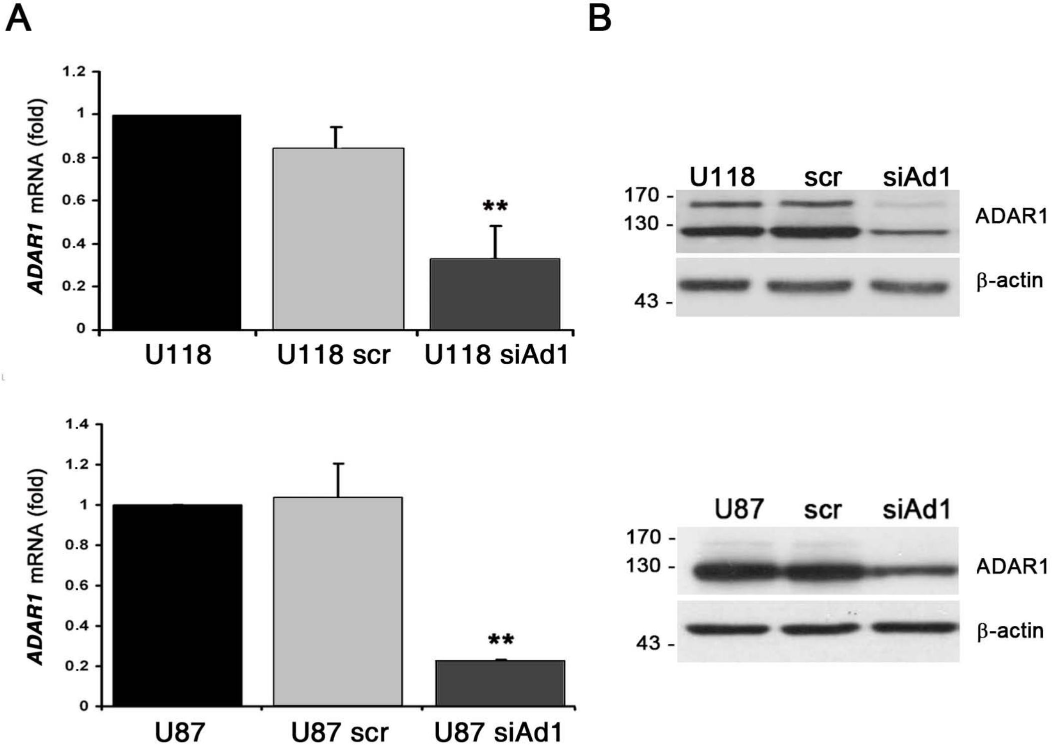

A
B
ADAR1 mRNA (fold)
1.2
1
0.8
0.6
0.4
0.2
0
**
U118
U118 scr
U118 siAd1
U118
scr
siAd1
170 -
130 -
43 -
ADAR1
β-actin
ADAR1 mRNA (fold)
1.4
1.2
1
0.8
0.6
0.4
0.2
0
**
U87
U87 scr
U87 siAd1
U87
scr
siAd1
170 -
130 -
43 -
ADAR1
β-actin

Fig. S3

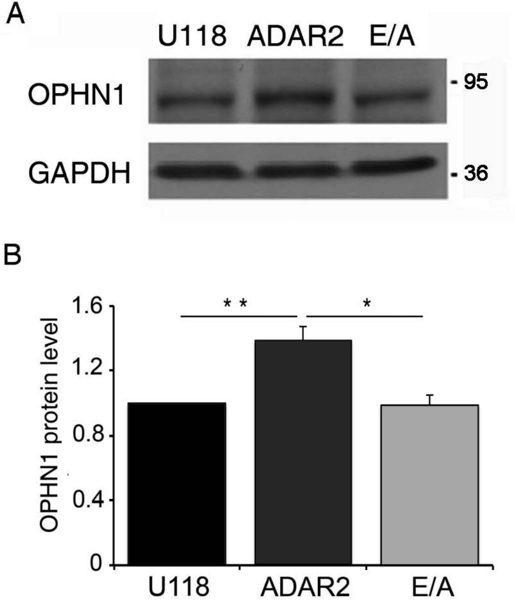
A
U118
ADAR2
E/A
OPHN1
GAPDH
- 95
- 36
B
OPHN1 protein level
1.6
1.2
0.8
0.4
0
**
*
U118
ADAR2
E/A

Fig. S4

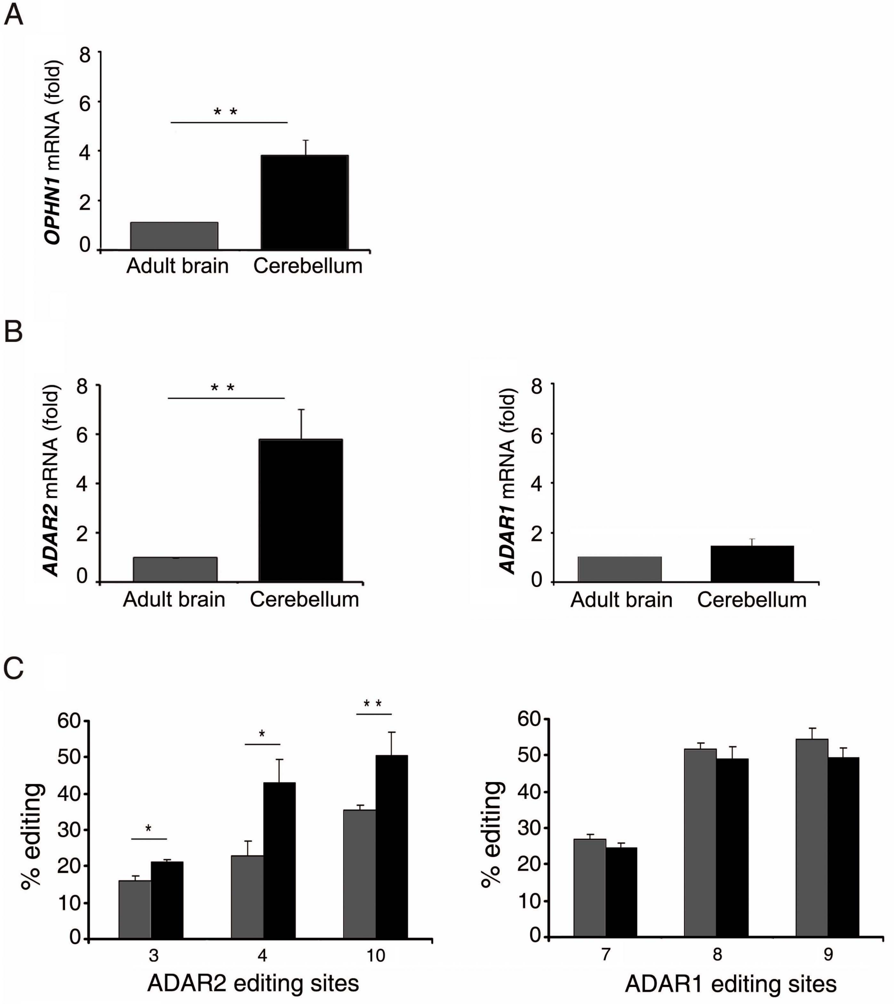
A
8
6
4
2
0
OPHN1 mRNA (fold)
* *
Adult brain
Cerebellum
B
ADAR2 mRNA (fold)
ADAR1 mRNA (fold)
C
60
50
40
30
20
10
% editing
*
* *
3
4
10
ADAR2 editing sites
7
8
9
ADAR1 editing sites

Fig. S5

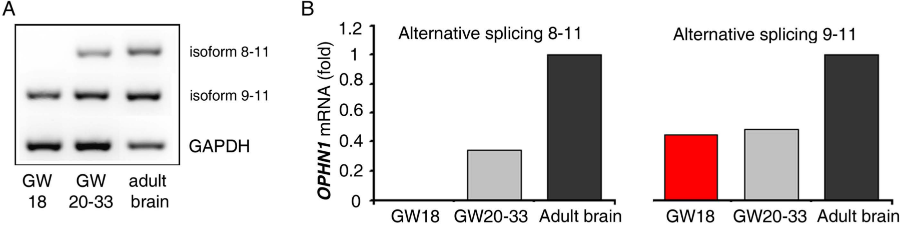
A
isoform 8-11
isoform 9-11
GAPDH
GW 18
GW 20-33
adult brain
B
Alternative splicing 8-11
Alternative splicing 9-11
OPHN1 mRNA (fold)
1.2
1
0.8
0.6
0.4
0.2
0
GW18
GW20-33
Adult brain
GW18
GW20-33
Adult brain

Fig. S6

Table S1

**OPHN1 - gDNA**	**Forward**	**Reverse**
Ex2 gDNA	5’-gtgcattgatgctctcgg-3’	5’-atttgcctcagagatcggg-3’
Ex3 gDNA	5’-cctgctcagagcacttacc-3’	5’-ccaagtgctctgtatccaca-3’
Ex4 gDNA	5’-gtctgtcaagcaccatgatta-3’	5’-gcaccaagaagctatctgac-3’
Ex5 gDNA	5’-aggaatcttgtatctgaac-3’	5’-gtcatgttagctgaggctac-3’
Ex6 gDNA	5’-gacagagttacaactacagac-3’	5’-ctgtttctaaggcatag-3’
Ex7 gDNA	5’-tggaccagaaactggaatcc-3’	5’-ttccactgattatccttcacg-3’
Ex8 gDNA	5’-agggattacaggcataagcc-3’	5’-ttggataggagtcagtaacc-3’
Ex9 gDNA	5’-tgcacatgctgtaagcaggg-3’	5’-aattcactgccgacaagtgg-3’
Ex10 gDNA	5’-gactctgaagagcaatgtgta-3’	5’-atggcctcacgtaacagtga-3’
Ex11 gDNA	5’-gacactgaagacactgtagg-3’	5’-ggacattgccaatttcacagc-3’
Ex12 gDNA	5’-tctgtaagcctatgcttgttc-3’	5’-actcatacactggcatatgct-3’
Ex13 gDNA	5’-cgcctggcctgttcaagta-3’	5’-cccatattcagacttgcgttag-3’
Ex14 gDNA	5’-ttaataggtgacacctgttgc-3’	5’-gcttgcataccactagtttag-3’
Ex15 gDNA	5’-atttggagagatgctgtagg-3’	5’-cacattttagcagtgtctcc-3’
Ex16 gDNA	5’-cagaccagaatgcagtcatg-3’	5’-cacgttaactccgctcaaca-3’
Ex17 gDNA	5’-agaatccaatacccagctgc-3’	5’-cttgagttagacagcaagcaa-3’
Ex18 gDNA	5’-gctcaatccagttgcccag-3’	5’-ttcctcacagagcattcagc-3’
Ex19 gDNA	5’-ttgtaatcttgccttccaatac-3’	5’-ggaagacaggtagtgagaata-3’
Ex20 gDNA	5’-ctagatataaggccaacaag-3’	5’-tggtcatgccaccgcttct-3’
Ex21 gDNA	5’-tgccacagtgacataactg-3’	5’-ctcagaaggatctcaagg-3’
Ex22 gDNA	5’-agccagagttgttagtcacc-3’	5’-gcctagtacaggacttcag-3’
Ex23 gDNA	5’-attggttctcacgtgtatacc-3’	5’-tgtatgctaaggatgcagttat-3’
Ex24 gDNA	5’-tagcactggcctagaagtgt-3’	5’-cgtaatgaaggcttatgtgga-3’

**OPHN1 - cDNA**	
cDNA-1Fw	5’-actgcgatagttagttagctctcc-3’
cDNA-1Rev	5’-ccgctccttggtgaag-3’
cDNA-2Fw	5’-ttgctgattaaacccttggaaaa-3’
cDNA-2Rev	5’-acatctcctgggcatttagg-3’
cDNA-3Fw	5’-agaacctatctaccacagcc-3’
cDNA-3Rev	5’-agtttccccactcctctgaa-3’
cDNA-4Fw	5’-ccctggatgaaagcgaagat-3’
cDNA-4Rev	5’-ctttcagaacctggaaaggg-3’
AluJo Fw	5’-acataaggccactaaggctc-3’
AluJo Rev	5’-gcctgagcaacatagtgaga-3’
AluSZ Fw	5’-aagtgatgggagagatagac-3’
AluSZ Rv	5’-cacaaccctgcctttactca-3’
AluJo 2Fw	5’-cccaactaacaagtgtcagg-3’
AluJo 2 Rev	5’-aacttagccaagtgtagtgg-3’

**OPHN1 - splicing**	
Splice exons 8-11 Fw	5’-cagtttacagaatgggccct-3’
Splice exons 9-11 Fw	5’-aagagaaatgggcccttgga-3’
Splice 12-13 Rev	5’-ggctgtggtagatggttct-3’
Exon 8 Fw	5’-agcctgtcttggcctttctt-3’
Exon 11 Rev	5’-ggttcctggcctttcattag-3’
