# Supplementary material for: Oligophrenin-1 (OPHN1), a Gene Involved in X-Linked Intellectual Disability, Undergoes RNA Editing and Alternative Splicing during Human Brain Development
Source: PLoS One. 2014 Mar 17;9(3):e91351. doi: 10.1371/journal.pone.0091351 (PMC3956665; doi:10.1371/journal.pone.0091351)
Supplement: Methods S1 — Includes Supporting Information Methods. (PDF) [file pone.0091351.s002.pdf]

Supporting Information Methods

Immunoblotting

Total protein extracts were isolated with RIPA lysis buffer (20 mM Tris-HCl (pH 7.5), 150 mM NaCl, 1 mM Na2EDTA, 1 mM EGTA, 1% NP-40, 1% sodium deoxycholate, 2.5 mM sodium pyrophosphate, 1 mM β-glycerophosphate) in the presence of a protease inhibitor mixture and phosphatase inhibitor cocktail (Sigma-Aldrich, St. Luis, MO, USA). Equal amounts of total cellular lysates (30 μg) were separated by SDS–PAGE, transferred on nitrocellulose membrane and analysed by immunoblotting with the appropriate antibodies and then revealed by ECL (GE Healthcare, Buckinghamshire, UK). The antibodies used in this study are: anti-OPHN1 (1:500, ProteinTech, Chicago, IL, USA), anti-ADAR1 (1:500, Sigma; 1:5000, polyclonal antibody [25]), anti-ADAR2 (1:200, Sigma), anti-β-actin (1:5000, Santa Cruz Biotechnology, Dallas, Texas, USA) and anti-GAPDH (1:5000, Cell Signaling, Danvers, MA, USA). The protein specific signals were quantified by densitometric analysis.

Semiquantitative RT-PCRs

The semiquantitative RT-PCRs were performed with Expand high fidelity Plus PCR System (Roche), using specific primers (0.2 μM each). PCR was conducted with the following parameters: 94 °C for 5 min, followed by 25 cycles 94 °C for 30 s, 56–60 °C for 30 s, and 72 °C for 1 min and by a final extension at 72 °C for 7 min. The *GAPDH* was chosen as internal control. The gene-specific primer pairs used for RT-PCR are described in Table S1 (OPHN1-splicing) in File S. The PCR products were run on a 1.5% agarose gel and the images acquired through the Gel-Doc apparatus (Bio-Rad) were analyzed using Quantity One software (Bio-Rad), in accordance with the manufacturer’s instructions.
